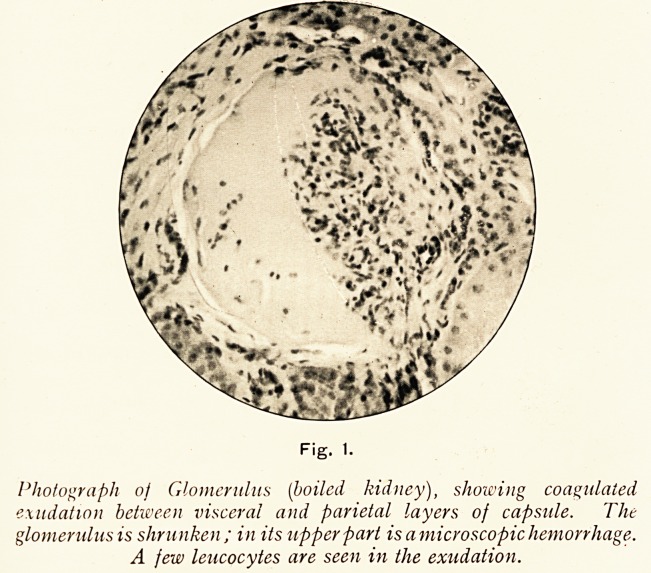# On the Renal Changes in a Case of Hemorrhage into the Pons with Consequent High Blood Pressure

**Published:** 1908-09

**Authors:** J. Michell Clarke

**Affiliations:** Professor of Medicine, University College, Bristol, and Physician to the Bristol General Hospital


### ON THE RENAL CHANGES IN A CASE OF HEMORRHAGE INTO THE PONS WITH CONSEQUENT HIGH BLOOD PRESSURE.

BY

J. MICHELL CLARKE, M.A., M.D., F.R.C.P.,

*Professor of Medicine, University College, Bristol, and Physician to the Bristol General Hospital.*

---

THE following case is interesting in relation to the changes which were found in the kidneys, presumably as the result of a sudden and great rise of blood pressure.

The patient, W.W., æt. 33, a dock labourer, was stated to have always been a healthy man, and never to have had any bad illness. He was at his work on the morning of November 20th, 1907, and felt some headache for half an hour. About 11 o'clock he suddenly fell down unconscious, and was brought to the Bristol General Hospital. The patient on admission was a strong, muscular man; he was profoundly unconscious and could not be roused. His face was pale, and the respiration shallow, slow and stertorous. Temperature 98.8°, the same on both sides of the body. Pulse 164, of high tension and incompressible. Pulses equal. Blood pressure on admission: systolic 250 mm. Hg., diastolic 200; taken later the systolic pressure was from 250 to 260 mm. Hg. Chest, well-developed, symmetrical; lungs, normal.

*Heart*, apex beat in 5th space, in nipple line or just outside it, with pulsation in epigastrium. Cardiac dulness; 3rd costal cartilage above, from right border of sternum to apex beat. No murmur. Aortic 2nd sound loud and accentuated.

*Abdomen.* The bladder was much distended, reaching above the umbilicus. Two quarts of urine were withdrawn by catheter.

*Nervous System.* The patient lay on his back—all four limbs were absolutely flaccid and motionless; when held up they dropped back on the bed. Both eyes were directed downwards and a little to the right; the pupils were small, but not pin-point, and did not react to light. The left naso-labial furrow was a little deeper than the right; there was slight spasm of the muscles of the right side of the neck. The abdominal, cremasteric and plantar reflexes were absent. The knee-jerks were active, the right greater than the left; the elbow and ankle-jerks were present. The breathing became gradually slower and more laboured, and the temperature steadily rose until it reached 110° at his death at 7.30 p.m. on the same evening. There were no convulsions at any time. The paralysis remained flaccid throughout. The urine withdrawn by catheter was neutral in reaction; sp. gravity 1010; it contained a large amount of proteid. This was found to consist almost entirely of albumose, with a small quantity of albumin. The urine was boiled with the addition of dilute acetic acid, and the coagulated albumin filtered off. The filtrate gave no further precipitate on boiling, but on half saturation with salicyl-sulphonic acid a dense precipitate formed, which cleared up on heating and reappeared on cooling. A similar precipitate was obtained with strong $HNO_3$ in the cold, and the biuret reaction was also present. The reaction for Bence-Jones albumin was not obtained. There was no sugar.

The *post-mortem* was made seventeen hours after death.

Body of a well-nourished, muscular man. The lungs were healthy, but showed intense congestion ; they were only slightly crepitant, but showed no actual consolidation. *Heart* weighed sixteen ounces. Both auriclea and the right ventricle were over-distended with blood ; both ventricles were thick-walled ; the muscle appeared healthy. At the base of the aorta, just above the valves, there was a small patch of commencing atheroma, probably due to syphilitic aortitis ; the aortic valves were opaque and slightly thickened, but held water. The coronary arteries were normal and pervious. *Abdomen :* Liver and stomach and intestines normal. Spleen weighed six ounces ; soft and dark. *Kidneys* showed intense congestion ; this was most marked in the cortex, but was also very pronounced in the medulla. In one kidney there was the scar of an old, small infarct. The capsules stripped readily, leaving a smooth surface. The consistence of the kidneys was normal, the cortex was somewhat swollen in relation to the medulla ; there was no evidence of interstitial change. The bladder was distended with urine. *Brain :* On removal of the calvarium and opening the dura, a large amount of dark, recent blood clot, which had been effused over the right hemisphere, escaped from the subdural space. On section the upper part of the pons was the seat of a large hemorrhage, which occupied the whole of the cross section of the pons, which it had completely destroyed except for a small shell of brain tissue surrounding the effused blood. No other lesion was found in the brain, which was entirely healthy except for the pontine hemorrhage.

Portions of the kidney were embedded in paraffin, cut and stained with hæmalum and eosin, by Van Gieson's stain, and with hæmatoxylin and rubin and rosin. Other portions were boiled at once, in order to fix, if possible, the proteid excreted in the urine. First, with the exception of the small, old infarct referred to above, the kidneys were the seat of acute changes, and showed no evidence of previous disease or of antecedent interstitial changes ; secondly, there was very general cloudy swelling of the tubular epithelium, due doubtless to the high temperature before death. The other changes found may be fairly attributed to the sudden and very high rise of blood pressure.

The boiled portions made good sections, and showed that numbers of the glomeruli contained a coagulable exudation in varying amount between the visceral and parietal layers of the capsular membrane ; in some cases the amount was so great as to effectively compress the glomerulus. (See Fig. 1.)

In addition, there were numerous microscopic hemorrhages into the glomeruli. These hemorrhages were mostly in the glomerulus itself, between the capillaries, often forming a thin layer spread over it, under or within the visceral layer of the capsule. In a very few the blood had escaped into the peri-

Fig. 1.

*Photograph of Glomerulus (boiled kidney), showing coagulated exudation between visceral and parietal layers of capsule. The glomerulus is shrunken; in its upper part is a microscopic hemorrhage. A few leucocytes are seen in the exudation.*

glomerular space, and in some instances a thin layer of blood lay under the visceral capsular layer, and coagulated exudation filled the glomerular space.

In the convoluted tubules there was so much swelling of the epithelium (boiled specimen) that no coagulated exudation could be distinctly made out in them; but many of the descending loops of Henle contained such an exudation.

The other sections (from non-boiled portions of kidney) showed most intense congestion of the organ throughout. The cortex from this cause appeared swollen, the vessels under the capsule intensely injected, also the capillaries of the glomeruli, from which minute hemorrhages had occurred as above stated; and there were also some minute hemorrhages into the interstitial tissue between the convoluted tubules. The general appearance of the cortex, in addition to these changes, appeared considerably altered, not altogether from the destruction of the individual cells of the tubules—which was a part of the process—as from a general disturbance or breaking-up of the normal arrangements of the various constituents of the kidney structure. (See Fig. 2.) In the medulla the straight tubules in many parts appeared crushed together by the injection of the vessels lying between them.

To sum up, in this case a man—with some slight thickening of the aortic valves not producing any symptoms and apparently in sound health, doing heavy work, and whose organs, examined after death, showed otherwise no signs of antecedent disease—is suddenly taken with a very large hemorrhage into the upper part of the Pons Varolii, dying a few hours afterwards.

The blood pressure about one and a half hours after the onset had already risen very high, and remained so until death. The temperature rose gradually, reaching 104° about five and a half hours after the onset and 110° at death. The sudden rise of blood pressure presumably caused the intense congestion of the kidneys, with the small hemorrhages into the glomeruli, mostly confined by the visceral layer of the glomerular capsules, which were found *post-mortem*; there was no hæmaturia. As the immediate effect, there was at once a very large excretion of urine containing albumose. From the appearances in sections of the boiled kidney the albumose was excreted through the glomeruli, and presumably as the effect of the rapid rise of blood pressure. That it was not due to damage of the cells of the glomeruli by hyperpyrexia is shown by the fact that two quarts of urine, containing albumose and albumin, were found in the bladder on admission to hospital, when the temperature was 99° only. It is interesting to compare this excretion of proteid in the form of albumose with the known fact that in the early stage of some cases of contracted granular kidney in which there is a sustained high blood pressure, albumose in small quantity is, for a long time, the form in which proteid is found in the urine. In addition, the high blood pressure seems to have produced a certain amount of disorganisation of the kidney structure.

In conclusion, it is my pleasing duty to thank Dr. E. V. Dunkley, Pathologist to the Hospital, for preparing excellent sections of the kidney.

### A CASE OF SEVERE TRIGEMINAL NEURALGIA SUCCESSFULLY TREATED BY EXCISION OF THE GASSERIAN GANGLION.

BY

ERNEST W. HEY GROVES, M.S., F.R.C.S.,

## Figures and Tables

**Figure f1:**
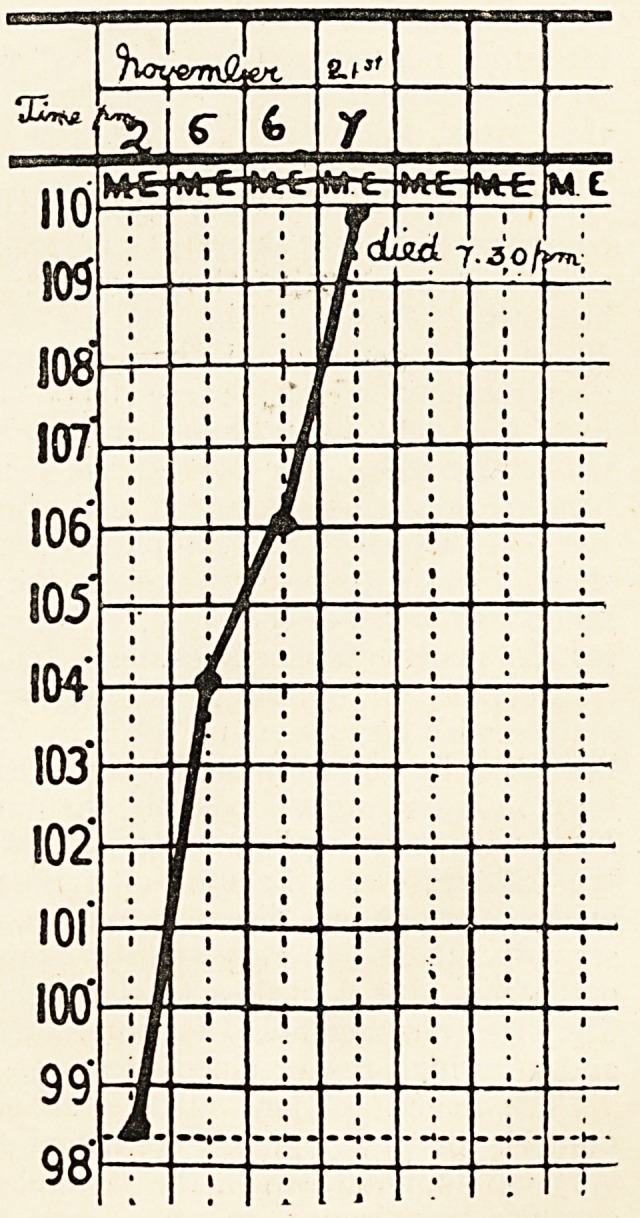


**Fig. 1. f2:**